# Short- and long-term outcomes after surgical treatment of 5918 patients with splenic flexure colon cancer by extended right colectomy, segmental colectomy and left colectomy: a systematic review and meta-analysis

**DOI:** 10.3389/fonc.2024.1244693

**Published:** 2024-04-15

**Authors:** Yu Cao, Mingze He, Kuo Chen, Zheng Liu, Denis I. Khlusov, Tatyana V. Khorobrykh, Xinren Cao, Polina D. Panova, Sergey K. Efetov, Airazat M. Kazaryan

**Affiliations:** ^1^ Department of Faculty Surgery No. 2, I.M. Sechenov First Moscow State Medical University, Moscow, Russia; ^2^ Institute for Urology and Reproductive Health, I.M. Sechenov First Moscow State Medical University, Moscow, Russia; ^3^ Department of Breast Surgery, The First Affiliated Hospital of Zhengzhou University, Zhengzhou, China; ^4^ Department of Colorectal Surgery, Cancer Hospital, Chinese Academy of Medical Science and Peking Union Medical College, Beijing, China; ^5^ Department of Gastrointestinal and Pediatric Surgery, Oslo University Hospital, Oslo, Norway; ^6^ Department of Surgery, Østfold Hospital Trust, Grålum, Norway; ^7^ Institute for Clinical Medicine, Medical Faculty, University of Oslo, Oslo, Norway; ^8^ Department of Surgery, Fonna Hospital Trust, Odda, Norway; ^9^ Department of Surgery No. 1, Yerevan State Medical University after M.Heratsi, Yerevan, Armenia

**Keywords:** splenic flexure colon cancer, colorectal surgery, extended right colectomy, segmental colectomy, left colectomysplenic flexure cancer, colon cancer, systematic review, meta-analysis

## Abstract

**Background:**

Colorectal cancer is among the most common cancers in the world, and splenic flexure colon cancer accounts for about 2-5% of them. There is still no consensus on the surgical treatment of splenic flexure colon cancer (SFCC), and the extent of surgical resection and lymph node dissection for SFCC is still controversial.

**Aim:**

To compare the postoperative and long-term oncologic outcomes of extended right colectomy (ERC), segmental colectomy (SC) and left colectomy (LC) for SFCC.

**Method:**

Up to March 2024, retrospective and prospective studies of ERC, SC, and LC for SFCC were searched through databases. Pooled weighted/standardized mean difference (WMD/SMD), odds ratio (OR) and hazard ratio (HR) with 95% confidence interval (CI) were calculated using a fixed effects model or random effects model, and meta-analysis was performed using Stata.

**Results:**

This meta-analysis includes 5,918 patients from 13 studies with more lymph node harvest (OR:6.29; 95%Cl: 3.66-8.91; Z=4.69, P=0), more operation time (WMD: 22.53; 95%Cl: 18.75-26.31; Z=11.68, P=0), more blood loss (WMD:58.44; 95%Cl: 20.20-96.68; Z=2.99, P=0.003), longer hospital stay (WMD:1.74; 95%Cl: 0.20-3.29; Z=2.21, P=0.03), longer time to return to regular diet (WMD:3.17; 95%Cl: 2.05-4.30; Z=5.53, P=0), longer first flatus time (WMD:1.66; 95%Cl: 0.96-2.37; Z=4.61, P=0) in ERC versus SC. More lymph node harvest (WMD: 3.52; 95% Cl: 1.59-5.44; Z=3.58, P=0) in ERC versus LC and LC versus SC (WMD: 1.97; 95% CI: 0.53-3.41; Z=2.68, P=0.007), respectively. There is no significant difference between anastomotic leakage, postoperative ileus, total postoperative complication, severe postoperative complication, wound infection, reoperations, R0 resection, postoperative mortality, 5-year overall survival (OS), 5-year disease-free survival (DFS) in three group of patients. In LC versus SC and ERC versus LC, there is no difference between operation time, blood loss, hospital stay, return to regular diet, and first flatus.

**Conclusion:**

In the included studies, SC and LC may be more advantageous, with fewer postoperative complications and faster recovery. ERC harvests more lymph nodes, but there is no significant difference in long-term OS and DFS between the three surgical approaches. Given that the included studies were retrospective, more randomized controlled trials are needed to validate this conclusion.

## 1 Introduction

Colorectal cancer (CRC) is one of the most commonly diagnosed cancers in the world. It has one of the highest rates of diagnosis and mortality of all cancer types, and the surgery-based combination therapy remains the treatment of choice for CRC (1, 2). The occurrence of colon cancer in splenic flexure is rare compared to other locations, accounting for merely 2-5% (3). Splenic flexure colon cancer (SFCC) is a tumor in the distal 1/3 of the transverse colon and the proximal 1/3 of the descending colon, which can be supplied with blood by the mid-colic and left colonic vessels due to its special feature of bidirectional blood supply (4, 5). In addition, lymphatic reflux can also flow to the superior and inferior mesenteric vessels. Due to the high degree of vascular variability, surgeons face uncertainty in determining the direction of the innervated vessels and lymphatic return. In addition, there are relatively few studies on the distribution of lymph node metastases and the value of lymph node dissection, with a high incidence of intestinal obstruction and postoperative complications in SFCC, as well as a low long-term survival rate (6). In contrast, colon adenocarcinoma of distal 2/3 descending colon, sigmoid colon and rectosigmoid junction origin, where the main lymphatic reflux is to the inferior mesenteric artery, is usually treated with standard left hemicolectomy or partial sigmoidectomy.

Extended right colectomy (ERC) is a common treatment for SFCC. Ligation of the ileocolic artery, the right colic artery, and the middle colic artery are needed, and depending on the location of the tumor, sometimes the left colon needs to be freed and the left colic artery ligated. Then, lymph nodes are dissected around the vessel, and finally, ileocolic anastomosis is performed. Other methods of treating SFCC include segmental colectomy (SC) and left colectomy (LC). SC requires ligation of the left branch of the middle colic artery and the left colic artery and colon-colon anastomosis of the remaining transverse colon and descending colon. LC requires high ligation of the inferior mesenteric artery and the left branch of the middle colic artery with transverse colon-rectal anastomosis. Because there is no consensus on the treatment of SFCC, the scope of surgical resection and lymph node dissection for splenic flexure carcinoma is still very controversial. This systematic review and meta-analysis focused on the surgical approach and the short- and long-term postoperative outcomes of SFCC.

## 2 Methods

### 2.1 Search strategy

This meta-analysis was performed according to the PRISMA 2020 (7) and AMSTAR (8) guidelines. Up to March 3, 2024, two independent researchers searched the PubMed, Embase, Scopus, and Cochrane Library databases using the keywords “splenic flexure colon cancer”, “extended right hemicolectomy”, “extended right colectomy”, “segmental colectomy”, “left hemicolectomy”, and “left colectomy” and the search strategy was changed accordingly according to the different database requirements. Relevant literature was also searched from the cited literature of related studies.

### 2.2 Inclusion and exclusion criteria

Studies were included if they conformed to the principle of PICO (S) [participants, interventions, comparisons, outcomes, (study design)].

Inclusion and exclusion criteria were as follows:

(1). Participants: Patients were diagnosed with colon cancer located at the splenic flexure or between the distal 3/1 of the transverse colon and the proximal 3/1 of the descending colon.
(2). Interventions: ERC, SC or LC performed by either laparoscopic or open approaches.
(3). Comparison: ERC versus SC or ERC versus LC or SC versus LC
(4). Outcome:
  - Primary outcome: anastomotic leakage, ileus, wound infection, total postoperative complications, severe postoperative complications, and reoperation.
  - Secondary outcomes: lymph node harvest, R0 resection, postoperative mortality, 5-year overall survival (OS), 5-year disease-free survival (DFS), operation time, blood loss, hospital stay, recovery to regular diet, and first flatus.
(5). Exclusion criteria were studies of other surgical techniques unrelated to the extent of SFCC resection and studies in which the extent of SFCC resection was not compared. In addition, review articles, letters, technical notes and books were excluded.

### 2.3 Data extraction and quality assessment

To assess the relevance of the retrieved studies, two reviewers independently screened their titles and abstracts, and all disagreements were resolved by discussion with a third reviewer. The following information was extracted from each included study: first author, year of publication, type of publication, count of patients, and patient characteristics (age, gender, body mass index, operation time, blood loss, hospital stay, recovery to regular diet, first flatus, anastomotic leakage, ileus, wound infection, total postoperative complication, severe postoperative complication, request reoperation, lymph node harvest, R0 resection, postoperative mortality, 5-year OS and 5-year DFS). To enhance sensitivity, certain reports were eliminated only if both reviewers eliminated specific reports. Later, the whole text within each study was examined, and these studies were included in this study during the screening process. The Newcastle−Ottawa Scale was used to ascertain the risk of bias, and reports achieving five or more stars were eligible (9). Randomized controlled trials used a tool made by the Cochrane Collaboration to examine the risk of bias (10).

### 2.4 Statistical analysis

STATA 17.0 was used to perform the statistical analysis. We used weighted mean differences (WMDs) and 95% confidence intervals (CIs) for continuous variables, whereas odds ratios (ORs) and 95% CIs were used for dichotomous variables. The random-effects model was implemented in this study to explore the statistical heterogeneity (I^2^>50% or P < 0.10). A fixed-effects model was implemented for further statistical analysis. Funnel plots and Egger’s test were performed to examine publication bias.

### 2.5 Information on the included studies

**Figure 1:**
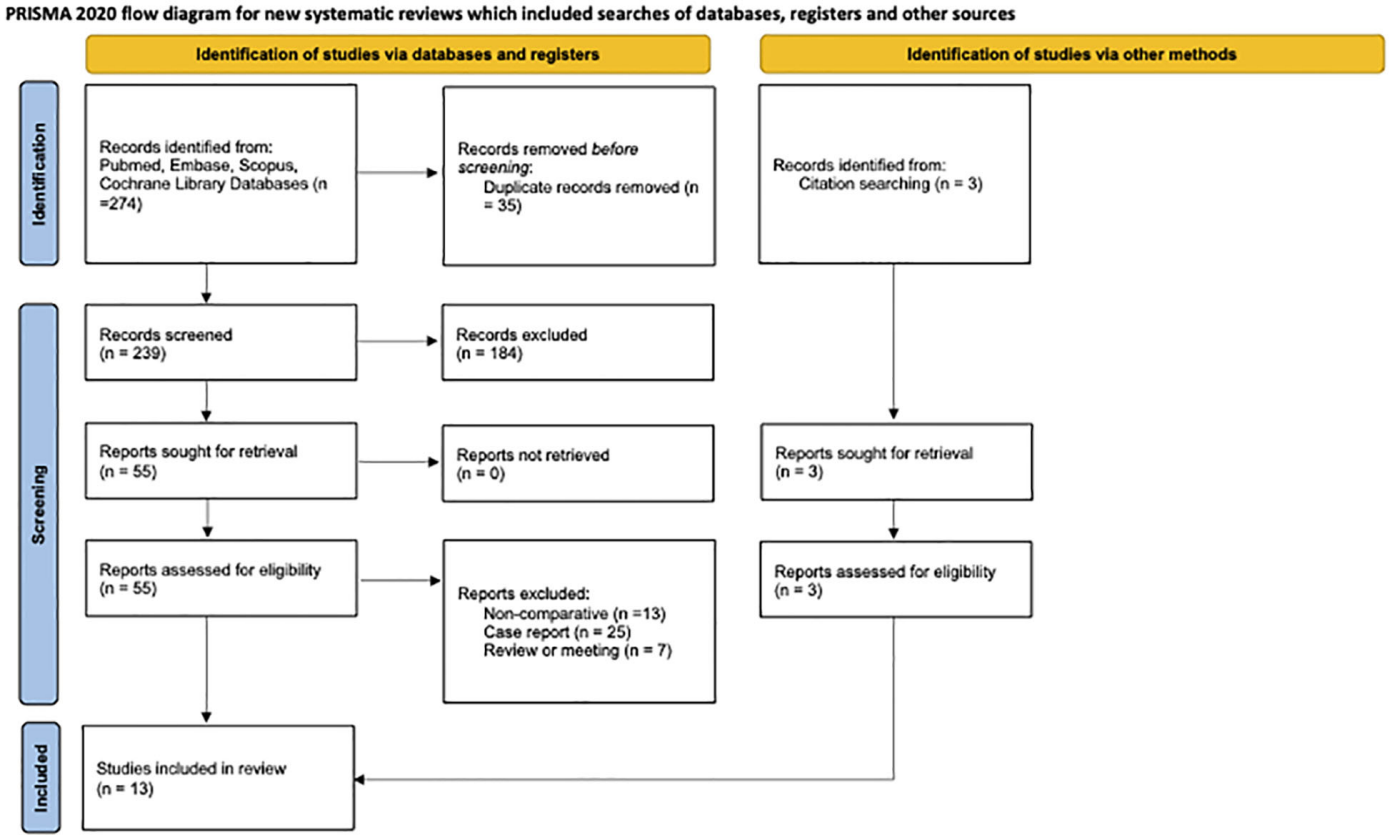
CONSORT flowchart.

The four databases yielded a total of 239 relevant studies; 3 studies identified through references list of the relevant articles. 35 studies were eliminated due to duplication, whereas the remaining literature was assessed by full-text assessment, and 13 studies were included. Among these, 13 were retrospective studies (6, 11–22) (**Figure 1**). In total, 5,918 splenic flexure cancer (SFC) patients were included, with 527 SFC patients in the ERC group, 1571 SFC patients in the SC group, and 3,820 SFC patients in the LC group. **Table 1** depicts the basic information about the patients and describes the Newcastle−Ottawa scale is shown in **Table 2**.

**Table 1:** The characteristic of included studies.

First author	Year	Country	comparison	Study design	Number of patients			Gender(M/F)			Age of patient		
					ERC	SC	LC	ERC	SC	LC	ERC	SC	LC
de’Angelis et al.	2021	Italy	ERC vs SC vs LC	RT	55	17	18	33/32	8/9	12/6	73(38-94)	74.7(43-84.3)	61.5(33-90.7)
Maurizio Degiuli et al.	2020	Italy	ERC vs SC vs LC	RT	100	791	413	57/43	445/346	244/169	67.3(11.9)	69.6(10.8)	67.7(11.0)
Refik Bademci et al.	2019	Spain	SC vs LC	RT	NA	28	55	NA	16/12	34/21	NA	68(11.7)	64(10.2)
El-Hendawy et al.	2022	Egypt	ERC vs SC vs LC	RT	40	40	40	26/14	26/14	26/14	55(29-80)	55(29-80)	55(29-80)
de’Angelis et al.	2020	France	ERC vs SC vs LC	RT	110	110	110	NA	NA	NA	68(35-91)	68.2(38-96.4)	68(44-94.2)
J. Martín Arévalo et al.	2018	Spain	ERC vs SC vs LC	RT	71	36	63	45/26	19/17	37/26	69(46-90)	70.5(37-90)	70.1(33-89)
M. Odermatt et al.	2013	UK	ERC vs LC	RT	38	NA	30	22/16	NA	13/17	74.5(42-95)	NA	71.5(51-86)
Allison J. Pang et al.	2022	Canada	SC vs LC	RT	NA	2550	499	NA	1397/1153	259/240	NA	64.8(13.0)	64.5(13.3)
Daniela Rega et al.	2019	Italy	ERC vs SC vs LC	RT	22	57	24	13/9	34/23	13/11	65(7.5)	67(10.5)	63.8(10.9)
Ji Hoon Kim et al.	2019	Korea	SC vs LC	RT	NA	118	236	NA	76/42	149/87	NA	59.8(12.6)	60.3(12.2)
de’Angelis et al.	2016	France	ERC vs LC	RT	27	NA	27	19/8	NA	19/8	66.8(9.3)	NA	65.5(10)
G Gravante et al.	2016	UK	ERC vs LC	RT	64	NA	34	37/27	NA	19/15	70.3(10.7)	NA	70(10.1)
Mingjin Huang et al.	2022	China	SC vs LC	RT	NA	73	22	NA	43/30	13/9	NA	56.6(14.5)	59.8(16.4)

ERC, extended right colectomy; SC, segmental colectomy; LC, left colectomy; RT, retrospective.

**Table 2:** Newcastle−Ottawa scale of included studies.

Study	Selection of the study groups	Comparability of the groups	Outcome	Total score
de’Angelis et al.	4	2	2	8
Maurizio Degiuli et al.	4	2	2	8
Refik Bademci et al.	4	2	2	8
El-Hendawy et al.	4	2	2	8
de’Angelis et al.	4	2	2	8
J. Martín Arévalo et al.	4	2	2	8
M. Odermatt et al.	4	2	2	8
Allison J. Pang et al.	4	2	2	8
Daniela Rega et al.	4	2	2	8
Ji Hoon Kim et al.	4	2	2	8
de’Angelis et al.	4	2	2	8
G Gravante et al.	4	2	2	8
Mingjin Huang et al.	4	2	2	8

## 3 Results

### 3.1 ERC versus SC

#### 3.1.1 Complications

*Anastomotic leakage*

Four studies with a total of 1266 patients compared anastomotic leakage between ERC and SC, with no significant differences between the two groups (OR: 0.91; 95% Cl: 0.47-1.78; Z=0.27, P=0.78), and no heterogeneity was observed; therefore, a random effects model was used (I^2 = ^0, P=0.64) (**Figure 2.1A**).

**Figure 2:**
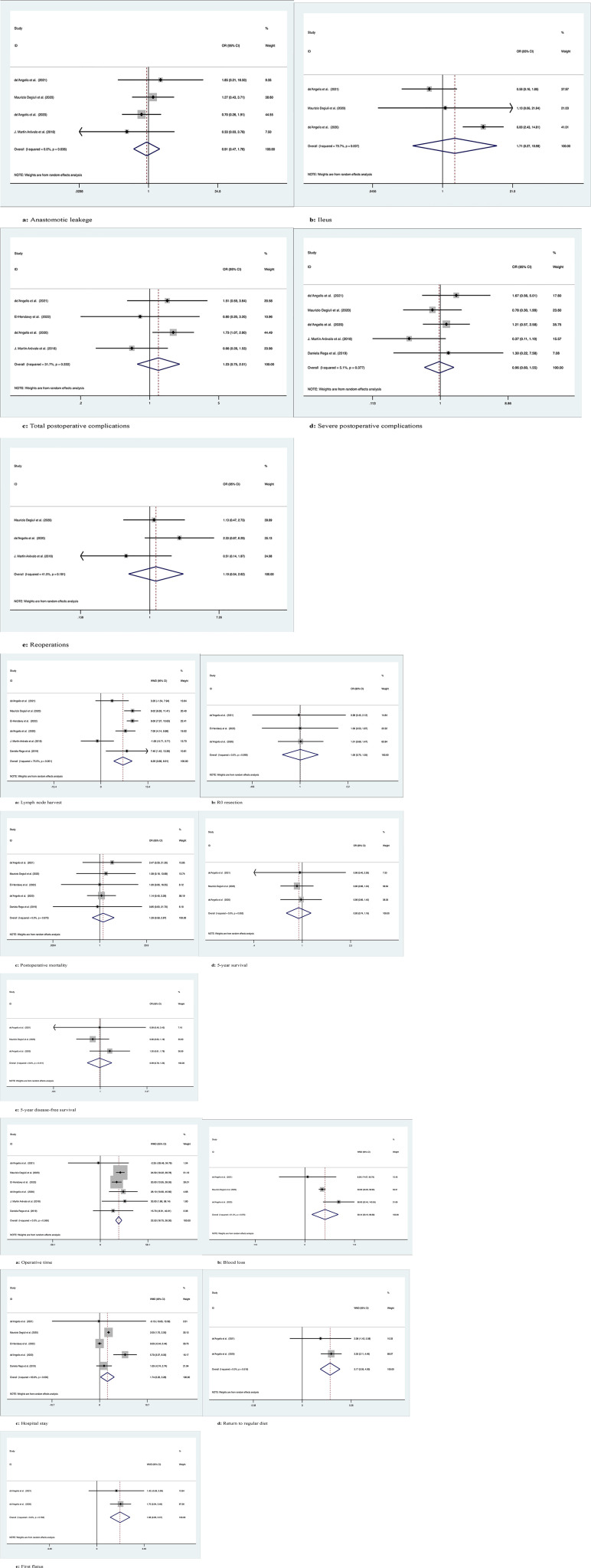
Meta-analytic graphs of extended right colectomy versus segmental colectomy. 1. **(A–E)** Complications; 2. **(A–E)** Pathology and long-term survival; 3. **(A–E)** Outcomes.

*Ileus*

The occurrence of postoperative ileus was recorded in a total of 1183 patients in 3 studies, with no significant difference between the two groups (OR: 1.71; 95% Cl: 0.27-10.98; Z=0.57, P=0.57), and high heterogeneity was observed using a random effects model (I^2 = ^79.7%, P=0.007) (**Figure 2.1B**).

*Total postoperative complications*

A total of 455 patients in four studies recorded the occurrence of total postoperative complications, with no significant difference between the two groups (OR: 1.23; 95% Cl: 0.75-2.01; Z=0.81, P=0.42), and low heterogeneity was observed, thus using a random effects model (I^2 = ^31.7%, P=0.22) (**Figure 2.1C**).

*Severe postoperative complications*

Five studies with a total of 1345 patients recorded the occurrence of serious postoperative complications (Clavien−Dindo grade ≥3), with no significant difference between the two groups (OR: 0.96; 95% Cl: 0.60-1.55; Z=0.16, P=0.88), due to the low heterogeneity observed, using a random effects model (I^2 = ^5.1%, P=0.38) (**Figure 2.1D**).

*Reoperations*

A total of 1218 patients in three studies were documented as requiring secondary surgery, with no significant difference between the two groups (OR: 1.19; 95% Cl: 0.54-2.62; Z=0.44, P=0.66), due to the low heterogeneity observed, using a random effects model (I^2 = ^41.5%, P=0.18) (**Figure 2.1E**).

#### 3.1.2 Pathology and long-term survival

*Lymph node harvest*

The number of lymph nodes harvested was recorded in a total of 1425 patients in six studies, with more lymph nodes harvested by ERC than by SC (OR: 6.29; 95% CI: 3.66-8.91; Z=4.69, P=0), and high heterogeneity was observed using a random effects model (I^2 = ^75.5%, P=0.001) (**Figure 2.2A**).

*R0 resection*

R0 resection was recorded in a total of 372 patients in the three studies, and there was no significant difference between the two groups (OR: 1.003; 95% CI: 0.75-1.35; Z=0.02, P=0.99). No heterogeneity was observed using a random effects model (I^2 = ^0, P=0.10) (**Figure 2.2B**).

*Postoperative mortality*

Postoperative mortality was recorded in a total of 1342 patients in five studies, with no significant difference between the two groups (OR: 1.29; 95% CI: 0.58-2.88; Z=0.63, P=0.53) and no heterogeneity observed using a random effects model (I^2 = ^0, P=0.97) (**Figure 2.2C**).

*5-year overall survival*

Three studies followed 1183 patients over time and recorded 5-year OS with no significant difference between the two groups (OR: 0.93; 95% CI: 0.74-1.18; Z=0.57, P=0.57), and no heterogeneity was observed using a random effects model (I^2 = ^0, P=0.95) (**Figure 2.2D**).

*5-year disease-free survival*

Three studies followed 1183 patients over time and recorded 5-year disease-free survival with no significant difference between the two groups (OR: 0.99; 95% Cl: 0.78-1.25; Z=0.12, P=0.91), and no heterogeneity was observed using a random effects model (I^2 = ^0, P=0.44) (**Figure 2.2E**).

#### 3.1.3 Outcomes

*Operation time*

Six studies recorded operative times in 1425 patients, with longer operative times for ERC than for SC (WMD: 22.53; 95% Cl: 18.75-26.31; Z=11.68, P=0), with no heterogeneity observed, using a random effects model (I^2 = ^0, P=0.57) (**Figure 2.3A**).

*Blood loss*

Intraoperative blood loss was recorded in 1183 patients in three studies, and more blood loss was observed with ERC than with SC (WMD: 58.44; 95% CI: 20.20-96.68; Z=2.99, P=0.003), with high heterogeneity observed using a random effects model (I^2 = ^61.2%, P=0.08) (**Figure 2.3B**).

*Hospital stay*

Five studies documented the length of postoperative hospital stay in 1342 patients, which was longer for ERC than for SC (WMD: 1.74; 95% CI: 0.20-3.28; Z=2.21, P=0.03), with high heterogeneity observed using a random effects model (I^2 = ^93.8%, P=0) (**Figure 2.3C**).

*Return to regular diet*

Two studies recorded the time to return to regular diet after surgery in 292 patients, with longer recovery times for ERH than for SC (WMD: 3.17; 95% CI: 2.05-4.30; Z=5.53, P=0), with no heterogeneity observed, using a random effects model (I^2 = ^0, P=0.52) (**Figure 2.3D**).

*First flatus*

Two studies recorded the time to postoperative first flatus in 292 patients, and the time to first flatus was longer for ERC than for SC (WMD: 1.66; 95% CI: 0.96-2.37; Z=4.61, P=0), with no heterogeneity observed, using a random effects model (I^2 = ^0, P=0.78) (**Figure 2.3E**).

### 3.2 LC versus SC

#### 3.2.1 Complications

*Anastomotic leakage*

Eight studies with a total of 5116 patients compared anastomotic leakage between LC and SC, with no significant differences between the two groups (OR: 0.97; 95% Cl: 0.68-1.38; Z=0.16, P=0.87) and no heterogeneity observed using a random effects model (I^2 = ^0, P=0.98) (**Figure 3.1A**).

**Figure 3:**
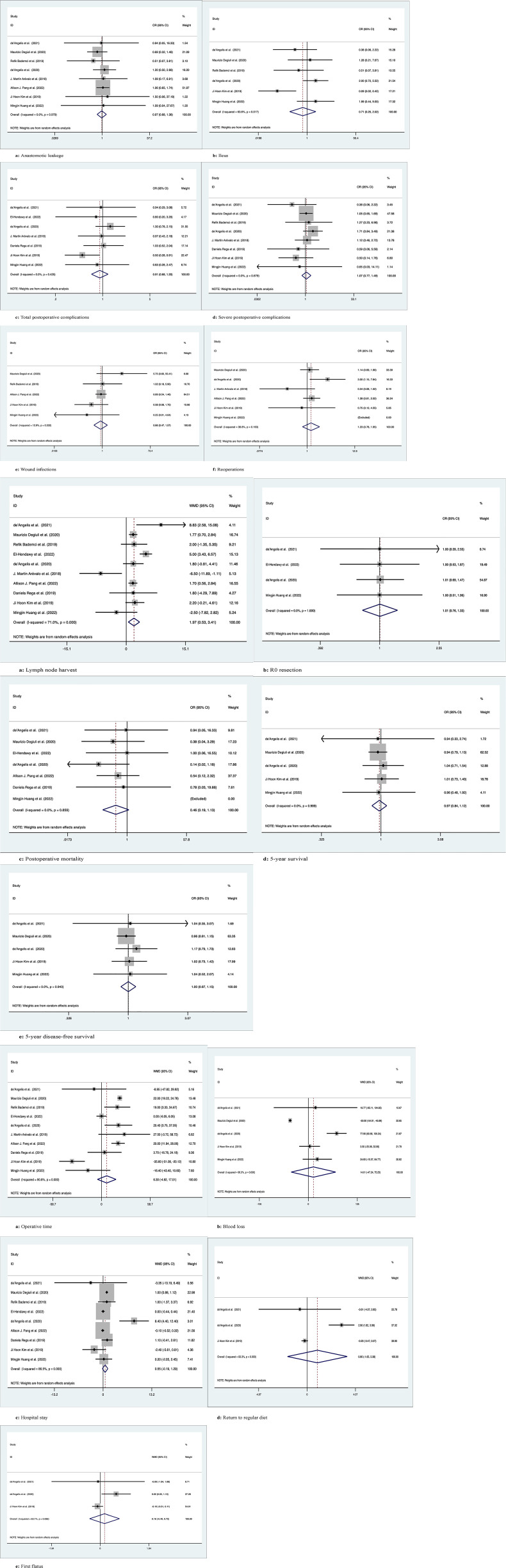
Meta-analytic graphs of left colectomy versus segmental colectomy. 1. **(A–F)** Complications; 2. **(A–E)** Pathology and long-term survival; 3. **(A–E)** Outcomes.

*Ileus*

A total of 1991 patients in six studies compared the occurrence of postoperative ileus, with no significant difference between the two groups (OR: 0.71; 95% CI: 0.25-2.02; Z=0.65, P=0.52), with high heterogeneity observed using a random effects model (I^2 = ^63.6%, P=0.02) (**Figure 3.1B**).

*Total postoperative complications*

Seven studies compared the occurrence of total postoperative complications in 941 patients, with no significant difference between the two groups (OR: 0.91; 95% CI: 0.68-1.20; Z=0.67, P=0.5), with low heterogeneity observed using a random effects model (I^2 = ^0, P= 0.43) (**Figure 3.1C**).

*Severe postoperative complications*

Eight studies documented severe postoperative complications (Clavien−Dindo grade ≥3) in 2148 patients, with no significant difference between the two groups (OR: 1.07; 95% Cl: 0.77-1.49; Z=0.4, P=0.69) and no heterogeneity observed using a random effects model (I^2 = ^0, P=0.68) (**Figure 3.1D**).

*Wound infection*

Five studies recorded postoperative wound infection in 4785 patients with no significant difference between the two groups (OR: 0.86; 95% CI: 0.47-1.57; Z=0.49, P=0.63) due to the low heterogeneity observed using a random effects model (I^2 = ^12.9%, P=0.33) (**Figure 3.1E**).

*Reoperations*

Six studies documented 5021 patients requiring reoperation after surgery, with no significant difference between the two groups (OR: 1.23; 95% CI: 0.78-1.95; Z=0.88, P=0.38), due to the low heterogeneity observed, using a random effects model (I^2 = ^38.8%, P=0.16) (**Figure 3.1F**).

#### 3.2.2 Pathology and long-term survival

*Lymph node harvest*

Ten studies counted the number of lymph nodes harvested in 5277 patients, with more lymph nodes harvested by LC than by SC (WMD: 1.97; 95% CI: 0.53-3.41; Z=2.68, P=0.007), due to the high heterogeneity observed, using a random effects model (I^2 = ^71%, P=0) (**Figure 3.2A**).

*R0 resection*

Four studies recorded R0 resection in 430 patients with no significant difference between the two groups (WMD: 1.01; 95% CI: 0.76-1.33; Z=0.04, P=0.97), and no heterogeneity was observed using a random effects model (I^2 = ^0, P=1) (**Figure 3.2B**).

*Postoperative mortality*

Seven studies recorded postoperative mortality in 4764 patients, with no significant difference between the two groups (WMD: 0.46; 95% CI: 0.19-1.13; Z=1.69, P=0.09) and no heterogeneity observed using a random effects model (I^2 = ^0, P=0.86) (**Figure 3.2C**).

*5-year overall survival*

Five studies followed and recorded 1908 patients for 5-year OS, with no significant difference between the two groups (OR: 0.97; 95% Cl: 0.84-1.12; Z=0.43, P=0.67) and no heterogeneity observed using a random effects model (I^2 = ^0, P=0.99) (**Figure 3.2D**).

*5-year disease-free survival*

Five studies followed and recorded 1908 patients for 5-year DFS, with no significant difference between the two groups (OR: 1.00; 95% Cl: 0.87-1.15; Z=0.03, P=0.98) and no heterogeneity observed using a random effects model (I^2 = ^0, P=0.94) (**Figure 3.2E**).

#### 3.2.3 Outcomes

*Operation time*

Ten studies counted operation time in 5277 patients, with no difference between the two groups (WMD: 6.30; 95% CI: -4.92-17.51; Z=1.1, P=0.27), and high heterogeneity was observed using a random effects model (I^2 = ^90.8%, P=0) (**Figure 3.3A**).

*Blood loss*

Five studies counted intraoperative blood loss in 1908 patients, with no significant difference between the two groups (WMD: 14.01; 95% CI: -47.24-75.25; Z=0.45, P=0.65), and high heterogeneity was observed using a random effects model (I^2 = ^96.3%, P=0) (**Figure 3.3B**).

*Hospital stay*

Nine studies recorded 5201 patients with no significant difference in postoperative hospital stay between the two groups (WMD: 0.55; 95% CI: -0.19-1.30; Z=1.45, P=0.15), with high heterogeneity observed using a random effects model (I^2 = ^86.5%, P=0) (**Figure 3.3C**).

*Return to regular diet*

Three studies recorded 609 patients who returned to a regular diet after surgery, with no significant difference between the two groups (WMD: 0.86; 95% CI: -1.65-3.38; Z=0.67, P=0.50), with high heterogeneity observed using a random effects model (I^2 = ^93.3%, P=0) (**Figure 3.3D**).

*First flatus*

Three studies recorded 609 patients with first postoperative flatus, with no significant difference between the two groups (WMD: 0.16; 95% CI: -0.40-0.72; z=0.57, p=0.57), with high heterogeneity observed using a random effects model (I2 = 63.1%, p=0.07) (**Figure 3.3E**).

### 3.3 ERC versus LC

#### 3.3.1 Complications

*Anastomotic leakage*

Seven studies recorded 1145 patients with postoperative anastomotic leakage, with no significant difference between the two groups (OR: 1.21; 95% Cl: 0.68-2.15; Z=0.66, P=0.51) and no heterogeneity observed using a random effects model (I^2 = ^0, P=0.67) (**Figure 4.1A**).

**Figure 4:**
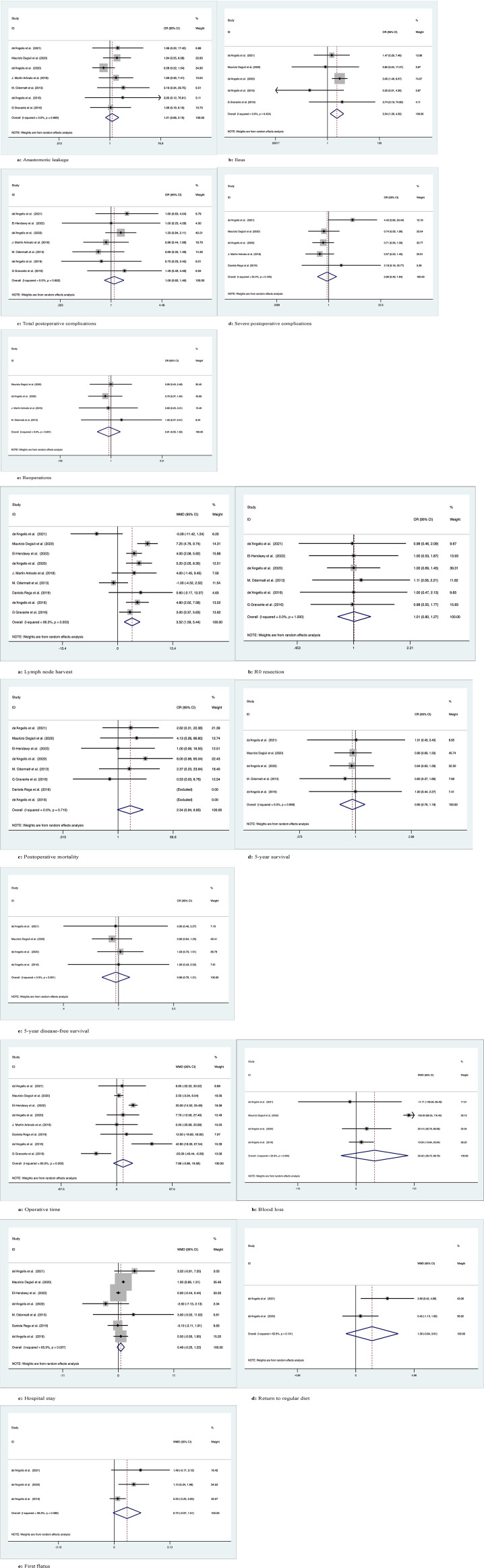
Meta-analytic graphs of extended right colectomy versus left colectomy. 1. **(A–E)** Complications; 2. **(A–E)** Pathology and long-term survival; 3. **(A–E)** Outcomes.

*Ileus*

Five studies recorded postoperative ileus in 958 patients, with more patients suffering from postoperative ileus with ERC than with LC (OR: 2.34; 95% CI: 1.28-4.30; Z=2.75, P=0.006), with no heterogeneity observed using a random effects model (I^2 = ^0, P=0.43) (**Figure 4.1B**).

*Total postoperative complications*

Seven studies recorded the occurrence of total postoperative complications in 712 patients, with no significant differences between the two groups (OR: 1.09; 95% CI: 0.82-1.46; Z=0.60, P=0.55) and no heterogeneity observed using a random effects model (I^2 = ^0, P=0.68) (**Figure 4.1C**).

*Severe postoperative complications*

Five studies documented the occurrence of severe postoperative complications (Clavien−Dindo grade ≥3) in 971 patients, with no significant difference between the two groups (OR: 0.90; 95% Cl: 0.49-1.64; Z=0.35, P=0.73), with low heterogeneity observed using a random effects model (I^2 = ^35.4%, P=0.19) (**Figure 4.1D**).

*Reoperations*

Four studies documented requiring reoperation after surgery in 935 patients, with no significant difference between the two groups (OR: 0.91; 95% CI: 0.55-1.50; Z=0.38, P=0.70) and no heterogeneity observed using a random effects model (I^2 = ^0, P=0.9) (**Figure 4.1E**).

#### 3.3.2 Pathology and long-term survival

*Lymph node harvest*

Nine studies recorded the number of lymph nodes harvested in 1271 patients, with more lymph nodes harvested for ERC than for LC (WMD: 3.52; 95% CI: 1.59-5.44; Z=3.58, P=0), with high heterogeneity observed using a random effects model (I^2 = ^66.3%, P=0.003) (**Figure 4.2A**).

*R0 resection*

Six studies recorded R0 resection in 593 patients with no significant difference between the two groups (WMD: 1.01; 95% CI: 0.80-1.27; Z=0.07, P=0.95), and no heterogeneity was observed using a random effects model (I^2 = ^0, P=1) (**Figure 4.2B**).

*Postoperative mortality*

Eight studies recorded postoperative mortality in 1152 patients, no significant difference between two groups of patients (OR: 2.54; 95% Cl: 0.94-6.85; Z=1.84, P=0.07), with no observed heterogeneity, using a random effects model (I^2 = ^0, P= 0.71) (**Figure 4.2C**).

*5-year OS*

Five studies followed and recorded 5-year OS in 928 patients over time, with no significant differences between the two groups (OR: 0.95; 95% Cl: 0.76-1.18; Z=0.47, P=0.64) and no heterogeneity observed using a random effects model (I^2 = ^0, P=1.0) (**Figure 4.2D**).

*5-year DFS*

Four studies followed and recorded 5-year DFS in 860 patients over time, with no significant differences between the two groups (OR: 0.96; 95% Cl: 0.76-1.21; Z=0.38, P=0.71) and no heterogeneity observed using a random-effects model (I^2 = ^0, P=0.96) (**Figure 4.2E**).

#### 3.3.3 Outcomes

*Operation time*

Eight studies recorded operation time in 1203 patients, with no significant difference between the two groups (WMD: 7.86; 95% CI: -3.86-19.58; Z=1.31, P=0.19), with high heterogeneity observed using a random effects model (I^2 = ^83%, P=0) (**Figure 4.3A**).

*Blood loss*

Four studies recorded intraoperative blood loss in 860 patients with no significant difference between the two groups (WMD: 35.02; 95% CI: -28.72-98.76; Z=1.08, P=0.28), with high heterogeneity observed using a random effects model (I^2 = ^94.8%, P=0) (**Figure 4.3B**).

*Hospital stay*

Seven studies documented the length of stay after surgery in 1054 patients, with no significant difference between the two groups (WMD: 0.48; 95% CI: -0.25-1.22; Z=1.30, P=0.19), with high heterogeneity observed using a random effects model (I^2 = ^65.9%, P=0.007) (**Figure 4.3C**).

*Return to regular diet*

Two studies recorded the time to return to regular diet after surgery in 293 patients, with no significant difference between the two groups (WMD: 1.39; 95% CI: -0.84-3.61; Z=1.22, P=0.22), with high heterogeneity observed using a random effects model (I^2 = ^62.8%, P=0.10) (**Figure 4.3D**).

*First flatus*

Three studies documented first postoperative flatus in 347 patients, no significant difference between two groups of patients (WMD: 0.72; 95% Cl: -0.07-1.51; Z=1.79, P=0.07), with high heterogeneity observed, using a random effects model (I^2 = ^58.8%, P=0.09) (**Figure 4.3E**).

The concise overview of the comparative outcomes after ERC, SC and LC are presented in **Table 3**.

**Table 3:** Comparison of outcomes between ERC, SC and LC pooled results.

Outcomes	ERC vs SC			LC vs SC			ERC vs LC		
	Number of patients (ERC/SC)	Summary measure (95%)	P	Number of patients (LC/SC)	Summary measure (95%)	P	Number of patients (ERC/LC)	Summary measure (95%)	P
Anastomotic leakage	315/951	OR: 0.91; 95% Cl: 0.47-1.78	0.78	1397/3719	OR:0.97; 95% Cl: 0.68-1.38	0.87	453/692	OR: 1.21; 95% Cl: 0.68-2.15	0.51
Postoperative ilues	265/918	OR: 1.71; 95% Cl: 0.27-10.98	0.57	854/1137	OR:0.70; 95% Cl: 0.25-2.02	0.52	356/602	OR:2.34; 95%Cl: 1.28-4.3	0.006
Total postoperative complication	255/200	OR:1.23; 95% Cl: 0.75-2.01	0.42	494/447	OR:0.91; 95%Cl: 0.68-1.20	0.5	393/319	OR: 1.09; 95%Cl: 0.82-1.46	0.55
Severe postoperative complication	337/1008	OR: 0.96; 95% Cl: 0.60-1.55	0.88	922/1226	OR: 1.07; 95% Cl: 0.77-1.49	0.69	346/625	OR: 0.90; 95% Cl: 0.49-1.64	0.73
Wound infection	NA	NA	NA	1225/3560	OR:0.86; 95%Cl: 0.47-1.57	0.63	NA	NA	NA
Reoperations	281/937	OR: 1.19; 95% Cl: 0.54-2.62	0.66	1343/3678	OR:1.23; 95%Cl: 0.78-1.95	0.38	319/616	OR: 0.91; 95%Cl: 0.55-1.50	0.7
Lymph node harvest	377/1048	OR:6.29; 95% Cl: 3.66-8.91	0	1461/3816	WMD:1.97; 95%Cl:0.53-3.41	0.007	515/756	WMD: 3.52; 95% Cl: 1.59-5.44	0
R0 resection	205/167	OR:1.003; 95%Cl: 0.75-1.35	0.99	190/240	WMD:1.005; 95%Cl: 0.76-1.33	0.97	334/259	WMD:1.01; 95%Cl: 0.80-1.27	0.95
Postoperative mortality	327/1015	OR:1.29; 95%Cl: 0.58-2.88	0.53	1126/3638	WMD:0.46; 95%Cl: 0.19-1.13	0.09	365/635	OR: 2.54; 95% Cl: 0.94-6.85	0.066
5-year OS	265/918	OR:0.93; 95%Cl: 0.74-1.18	0.57	799/1109	OR:0.97; 95% Cl: 0.84-1.12	0.67	330/598	OR: 0.95; 95% Cl: 0.76-1.18	0.64
5-year DFS	265/918	OR:0.99; 95% Cl: 0.78-1.25	0.91	799/1109	OR:1.00; 95% Cl: 0.87-1.15	0.98	292/568	OR: 0.96; 95% Cl: 0.76-1.21	0.71
Operation time	377/1048	WMD:22.53; 95%Cl:18.75-26.31	0	1461/3816	WMD:6.30; 95%Cl: -4.92-17.51	0.27	477/726	WMD:7.86; 95%Cl: -3.86-19.58	0.19
Blood loss	265/918	WMD:58.44; 95%Cl: 20.20-96.68	0.003	799/1109	WMD:14.00; 95%Cl: -47.24-75.25	0.65	292/568	WMD:35.02; 95%Cl: -28.72-98.76	0.28
Hospital stays	327/1015	WMD:1.74; 95%Cl: 0.20-3.29	0.03	1417/3784	WMD:0.55; 95% Cl: -0.19-1.30	0.15	392/662	WMD:0.48; 95%Cl: -0.25-1.22	0.19
Return to regular diet	165/127	WMD:3.17; 95%Cl: 2.05-4.30	0	364/245	WMD:0.86; 95%Cl: -1.65-3.38	0.5	165/128	WMD:1.39; 95%Cl: -0.84-3.61	0.22
First flatus	165/127	WMD:1.66; 95%Cl: 0.96-2.37	0	364/245	WMD:0.16; 95%Cl: -0.40-0.72	0.57	192/155	WMD:0.72; 95% Cl: -0.07-1.51	0.073

## 4 Discussion

We performed a systematic review and meta-analysis after screening 13 articles with a total of 5918 patients to compare postoperative complications and long-term survival between ERC, SC, and LC. According to the results we observed, it is explainable that there is currently no consensus among the three surgical approaches to treat SFCC, as there are no significant differences in critical indicators such as total postoperative complications, severe postoperative complications, anastomotic leakage, 5-year OS and 5-year DFS rates. For the requirement of reoperation and R0 resection, a lack of significant differences among the three groups was also observed.

Compared with ERC, less surgical manipulation might be the evidence of shorter operative time, less intraoperative blood loss, shorter hospital stays, faster return to regular diet, and shorter time to first flatus for SC. ERC is associated with a higher occurrence of postoperative ileus than LC, higher postoperative mortality, and shorter time to first flatus after surgery. In terms of lymph node harvest, the greater number of lymph nodes retrieved in ERC is reasonable, as a larger extent of surgical resection is performed in case of ERC than in case of LC and SC, while the greater number of vessels that required ligation for ERC is also considered one of the possible reasons. However, the number of lymph nodes harvested in LC was greater than that harvested in SC, which might be speculated to be because the tumor location and the level of invasion differ and lymph node distribution varies between individuals. As we understand, the bidirectional blood supply of the splenic flexure colon from the middle colic artery (MCA) and left colic artery (LCA) is complicated. Nearly 30% of patients also have the accessory MCA derived from the celiac trunk (23, 24). According to a study conducted by Fukuoka et al. (24), the blood supply of the splenic flexure colon is highly individualized and is thereby subdivided into 6 categories, among which the LCA is the most prominent artery to supply the splenic flexure colon. Surprisingly, there was also no blood supply to the splenic flexure colon in nearly 25% of patients. As a result, the subsequent treatment plan based on the unique blood supply should be taken into consideration. Despite the lack of relevant literature resources, we may boldly suspect that the oncological outcome is also correlated with individual anatomical variations. However, the statistical support is inadequate, therefore, further detailed studies should be carried out to facilitate the optimal surgical technique selection by surgeons, which is also in the scope of personalized medicine (25).

The 5-year survival rate for lymph node-negative patients is 70%-80% compared to 30%-60% for lymph node-positive patients (26). Hence, a precise surgical tactic of blood vessel ligation and lymph node management should be taken into consideration to minimize the occurrence of hematological and lymphatic dissemination. It has been suggested to use indocyanine green (ICG) to navigate lymph node dissection. Meanwhile, near-infrared imaging (NIR) for clear vessel visualization may also contribute to precise vessel ligation (23).

Postoperative anastomotic leakage remains a serious complication that is more commonly seen during emergency operations than during elective operations. Logically, post ileo-colonic anastomosis leakage tends to be prominent due to the difference in the diameter of the colon and ileum (27, 28). However, it is interesting to determine that the three groups of anastomoses did not show any significant differences. Different positions of the tumor and the experience of the surgeon might lead to such outcomes, but further consideration and evaluation are still of clinical value. However, the bulk of the researchers did not include emergency scenarios in their data analysis despite the fact that ERC is the preferred procedure in an emergency setting with intestinal obstruction (29). In our study, only 287 cases among 5918 cases underwent emergency operations, making up only 4.85% of the total cases. The preoperatively extensive assessment of surgical technique selection is insufficient for emergency surgery when compared to the elective surgery of SFC. As a result, it can have an impact on the primary outcome and lead to complications, which might ultimately result in bias. The thorough exploration and examination of the three independent surgical procedures based on the emergency condition, however, requires high-quality and satisfactory prospective studies and randomized clinical trials (RCTs) due to the dearth of RCTs specifically focused on the three approaches during emergency surgery.

In addition to the 5-year survival rate as the most important indicator, lymph node harvest and anastomosis leakage with detailed anastomosis type selection should also be deeply studied as the primary endpoints. Developing novel surgical approaches for facilitating surgical resection and enhancing long-term survival is still meaningful. Based on our study, the advantages and shortcomings of the three surgical techniques are still under-investigated. The determination of any one of them for surgical intervention of splenic flexure colon cancer seems reasonable, and consensus has still not been reached. Because limited amounts and qualities of samples are included in our study, the conclusion we made is still robust and well documented. Currently, the observational studies available are not sufficient to provide more encouraging findings, thus additional clinical trials incorporating a meticulous comparison of ERC, SC, and LC with solid statistical support are anticipated.

Moreover, type 2 error, publication bias and reporting bias could not be avoided, as the sample sizes were inadequate, which requires further estimation.

## 5 Conclusion

In observational studies, SC appeared to have a more favorable outcome intraoperatively and postoperatively, but ERC harvested more lymph nodes. There was no obvious difference among the three surgical modalities in long-term survival, and considering that the included were all retrospective studies, more randomized controlled studies are needed to confirm this conclusion.

## Data availability statement

The original contributions presented in the study are included in the article/supplementary material. Further inquiries can be directed to the corresponding author.

## Author contributions

YC, MH, KC, ZL, DK, TK, XC, PP, SE, AK conceptualized the study. YC, MH, KC, and ZL performed the literature analysis and wrote different parts of the original manuscript draft. DK, TK, and XC Processed data. PP, SE, and AK revised, edited, and extended the final draft. All authors have reviewed and approved the manuscript before submission.
